# Supplementary material for: Correlation of Genotype-Phenotype of Congenital Hypothyroidism Cohort Diagnosed by Newborn Screening: A Long-Term Observational Study
Source: Int J Neonatal Screen. 2025 Oct 20;11(4):98. doi: 10.3390/ijns11040098 (PMC12550986; doi:10.3390/ijns11040098)
Supplement: Supplementary file 1 [file IJNS-11-00098-s001.zip › IJNS-3862892-supplementary.pdf]

Table S1 The Mode of Inheritance with CH-Related Genes

Gene	OMIM	Mode of inheritance
NKX2-1	600635	AD
POU1F1	173110	AD, AR
PAX8	167415	AD
NKX2-5	600584	AD
TBL1X	300196	X-linked
JAG1	601920	AD
TSHR	603372	AD, AR
GNAS	139320	AD
SLC5A5	601843	AR
SLC26A4/PDS	605646	AR
DUOX2	606759	AD, AR
DUOXA2	612772	AR
TPO	606765	AR
TG	188450	AR

Abbreviations: AD, autosomal dominant; AR, autosomal recessive; OMIM, Online Mendelian Inheritance in Men (https://www.ncbi .nlm.nih.gov/omim/).

Table S2 Differences in Overall Characteristics Between "*DUOX2* Monoallelic Mutations" and "*DUOX2* Biallelic Mutations" Groups

Characteristic	Case (%) Monoallelic variation (n=5)	Biallelic variation (n=23)	*p* value[a]
Male	4（80.0）	12（52.2）	0.355
TSH level at NBS, M (Q1, Q3), mIU/L	100.00(70.21,275.96)	100.00(79.40,100.00)	0.348
FT4 level at diagnosis, M (Q1, Q3), pmol/L	2.93(2.57,6.11)	3.90(1.15,6.50)	0.908
L-T4 dose at 6 mo, mean (SD), µg/kg·d	4.10(1.71)	3.73(2.00)	0.491
L-T4 dose at 12 mo, mean (SD), µg/kg·d	3.69(1.65)	3.04(1.01)	0.401
L-T4 dose at 18 mo, mean (SD), µg/kg·d	3.52(1.56)	3.39(2.45)	0.497
L-T4 dose at 24 mo, mean (SD), µg/kg·d	3.04(1.47)	2.48(1.55)	0.443
outcome			0.666
PCH	4(80.0)	17(74.0)	
TCH	1(20.0)	3(13.0)	
Undiagnosed	0(0)	3(13.0)	

Abbreviations: TSH, thyroid stimulating hormone; NBS, newborn screening; FT4, free thyroxine; L-T4, levothyroxine; PCH, permanent congenital hypothyroidism; TCH, transient congenital hypothyroidism; NA, not applicable.

[a] P value from χ^2 test for categorical variables and Mann-Whitney *U* test for continuous variables; unknown values were excluded.

Table S3 Differences in the Overall Characteristics Between "Negative", "Monogenic Mutations" and "Oligogenic Mutations" Groups.

Characteristic	Negative (n=52)	Monogenic variation (n=54)	Oligogenic variation (n=13)	*p* value[a]
Male n(%)	16（30.8）	26（83.3）	5（38.5）	0.541
TSH level at NBS, M (Q1, Q3), mIU/L	148.86(100.00,200.13)	112.00(98.03,171.21)	150.00(92.31,221.61)	0.346
FT4 level at diagnosis, M (Q1, Q3), pmol/L	4.40(1.38,7.31)	3.08(1.09,5.50)	1.78(0.77,5.15)	0.220
L-T4 dose at 6 mo, mean (SD), μg/kg·d	4.61(2.11)	4.41(2.08)	4.44(2.15)	0.821
L-T4 dose at 12 mo, mean (SD), μg/kg·d	3.59(1.61)	3.39(1.12)	3.82(1.48)	0.545
L-T4 dose at 18 mo, mean (SD), μg/kg·d	4.11(1.60)	3.53(1.83)	3.49(1.49)	0.370
L-T4 dose at 24 mo, mean (SD), μg/kg·d	4.17(1.87)	3.08(1.42)	3.56(1 .69)	0.155
Thyroid ultrasound n(%)				0.001
Hypoplasia	16(30.8)	5(9.3)[bc]	7(53.8)	
Normal	25(48.1)	34(63.0)	6(46.2)	
Enlarged	5(9.6)[d]	19(35.2)	2(15.4)	
Outcome n(%)				0.014
PCH	28(53.8)[ef]	48(88.9)	12(82.3)	
TCH	2(3.8)	4(7.7)	2(15.4)	
Undiagnosed	16(30.8)[g]	6(11.1)	1(7.7)	

Abbreviations: TSH, thyroid stimulating hormone; NBS, newborn screening; FT4, free thyroxine; L-T4, levothyroxine; PCH, permanent congenital hypothyroidism; TCH, transient congenital hypothyroidism.

[a] P value from χ^2 test for categorical variables and Kruskal-Wallis test for continuous variables; unknown values were excluded.

Significant pairwise comparisons after Dunn test with Bonferroni correction are indicated below the overall *P* values. [b] *Negative* vs *monogenic*: P=0.005. [c] *monogenic* vs *oligogenic*: P=0.027. [d] *Negative* vs *monogenic*: P=0.002. [e] *Negative* vs *monogenic*: P<0.001. [f] *monogenic* vs *oligogenic*: P=0.01. [g] *Negative* vs *monogenic*: P=0.013.

Table S4 Classification of Gene Variants Based on ACMG Guidelines

Gene	Transcript	Variant	Classification	Evidence
JAG1	NM_000214.3	c.574T>C	LP	PP3_Strong; PP2; PM2_Supporting; PM1;
TSHR	NM_000369.5	c.1195delA	LP	PVS1_Unset; PM2_Supporting; PP4
TSHR	NM_000369.5	c.1291G>C	LP	PP3_Strong; PM2_Supporting; PM1;
TSHR	NM_000369.5	c.1349G>A	LP	PS3_Supporting; PP3_Strong; PM1;
TSHR	NM_000369.5	c.154C>A	LP	PS3_Supporting; PM2_Supporting；PP3
TSHR	NM_000369.5	c.1580T>A	LP	PP3_Strong; PM2_Supporting; PM1;
TSHR	NM_000369.5	c.1736C>T	LP	PM2_Supporting; PM1; PM3, PP4
TSHR	NM_000369.5	c.1942A>T	LP	PM2_Supporting; PM1; PM3, PP4
TSHR	NM_000369.5	c.243-1G>C	P	PVS1_Unset; PM2_Supporting; PM3
TSHR	NM_000369.5	c.350T>G	LP	PP3_Moderate; PM2_Supporting; PM1; PP4
TSHR	NM_000369.5	c.394G>C	LP	PS3_Supporting; PP3_Moderate; PM1; PP4
TSHR	NM_000369.5	c.463A>T	LP	PM2_Supporting; PM1; PM3, PP4
SLC26A4	NM_000441.2	c.1003T>C	LP	PS3_Supporting; PM2_Supporting；PP3
SLC26A4	NM_000441.2	c.1790T>A	LP	PVS1_VeryStrong; PP3_Strong; PM2_Supporting;
SLC5A5	NM_000453.3	c.1572T>G	LP	PVS1_Unset; PP3_Strong; PM2_Supporting; PP4
SLC5A5	NM_000453.3	c.206C>T	LP	PP3_Strong; PM2_Supporting; PM1; BP1;
TPO	NM_000547.6	c.1101_1110del	P	PVS1_Unset; PM2_Supporting; PM3
TPO	NM_000547.6	c.1229G>A	LP	PVS1_Unset; PP3_Strong; PM2_Supporting; PP4
TPO	NM_000547.6	c.1246C>T	LP	PP3_Moderate; PM2_Supporting; PM1; PP4
TPO	NM_000547.6	c.1428_1448del	P	PM4; PM2_Supporting; PM3, PP4
TPO	NM_000547.6	c.1781G>A	LP	PVS1_Unset; PP3_Strong; PM2_Supporting; PP4
TPO	NM_000547.6	c.1905G>C	LP	PP3_Moderate; PM2_Supporting; PM1; PP4
TPO	NM_000547.6	c.1949G>A	LP	PP3_Strong; PM2_Supporting; PM1;
TPO	NM_000547.6	c.2306G>A	LP	PM2_Supporting; PM1; PM3; PP4
TPO	NM_000547.6	c.2387A>G	LP	PP3_Moderate; PM2_Supporting; PM1; PP4
TPO	NM_000547.6	c.2473T>C	LP	PP3_Moderate; PM2_Supporting; PM1; PP4
TPO	NM_000547.6	c.256G>A	LP	PM2_Supporting; PM1; PM3, PP4
TPO	NM_000547.6	c.2578G>A	LP	PS3_Supporting; PP3_Moderate; PM2_Supporting; PP4
TPO	NM_000547.6	c.2647C>T	LP	PS3_Supporting; PM2_Supporting；PP3
TPO	NM_000547.6	c.2677G>A	LP	PM2_Supporting; PM1; PM3, PP4
TPO	NM_000547.6	c.425G>A	LP	PP3_Strong; PM2_Supporting; PM1;

TPO	NM_000547.6	c.551G>A	LP	PP3_Strong; PM2_Supporting; PM1;
TPO	NM_000547.6	c.612G>A	LP	PS3_Supporting; PM2_Supporting；PP3
TPO	NM_000547.6	c.872G>A	LP	PP3_Strong; PM2_Supporting; PM1;
TPO	NM_000547.6	c.940C>T	LP	PP3_Strong; PM2_Supporting; PM1;
NKX2-1	NM_001079668.3	c.1054G>A	LP	PS3_Supporting; PM2_Supporting；PP3
GNAS	NM_000516.7	c.302_303del	LP	PM2_Supporting; BP4_Supporting;
GNAS	NM_001309840	c.946G>C	LP	PP3_Moderate; PM2_Supporting; PM1; PP4
TG	NM_003235	c.1010G>A	LP	PVS1_Unset; PP3_Strong; PM2_Supporting; PP4
TG	NM_003235	c.2359C>T	LP	PVS1_Unset; PP3_Moderate; PM2_Supporting; PP4
TG	NM_003235	c.2963G>A	LP	PS3_Supporting; PP3_Strong; PM1;
TG	NM_003235	c.4783delG	LP	PVS1_Unset; PM2_Supporting; PP4
TG	NM_003235	c.4859C>T	LP	PS3_Supporting; PP3_Moderate; PM1; PP4
TG	NM_003235	c.5386-5387delCAinsGCAATATCTT	P	PM2_Supporting; PVS1
TG	NM_003235	c.5928A>G	LP	PM2_Supporting; PM1; PM3, PP4
TG	NM_003235	c.6517C>T	LP	PVS1_Unset; PP3_Strong; PM2_Supporting; PP4
TG	NM_003235	c.6707C>T	LP	PP3_Moderate; PM2_Supporting; PM1; PP4
TG	NM_003235	c.7198A>G	LP	PM2_Supporting; PM1; PM3, PP4
TG	NM_003235	c.7733G>A	LP	PM2_Supporting; PM1; PM3, PP4
TG	NM_003235	c.8119C>T	LP	PVS1_Unset; PP3_Moderate; PM2_Supporting; PP4
PAX8	NM_003466.4	c.1046C>A	LP	PP3_Supporting; PM2_Supporting; PP4; PM6
NKX2-5	NM_004387	c.641_642insGCC	LP	PM2_Supporting; PS4；PP4;
DUOX2	NM_014080.5	c.1087A>G	LP	PM2_Supporting; PS4；PP4;
DUOX2	NM_014080.5	c.1268C>T	LP	PS3_Supporting; PM2_Supporting；PP3
DUOX2	NM_014080.5	c.1295G>A	LP	PS3_Supporting; PM2_Supporting；PP3
DUOX2	NM_014080.5	c.1300C>T	LP	PVS1_Unset; PP3_Strong; PM2_Supporting; PP4
DUOX2	NM_014080.5	c.1310G>C	LP	PP3_Moderate; PM2_Supporting; PM1; PP4
DUOX2	NM_014080.5	c.1588A>T	LP	PVS1_Unset; PS3_Supporting; PP3_Strong; PP4
DUOX2	NM_014080.5	c.160+5G>A	P	PVS1_Unset; PM2_Supporting; PM3
DUOX2	NM_014080.5	c.1873C>T	LP	PVS1_Unset; PP3_Strong; PM2_Supporting; PP4
DUOX2	NM_014080.5	c.2048G>T	LP	PS3_Supporting; PM2_Supporting；PP3
DUOX2	NM_014080.5	c.2182G>A	LP	PS3_Supporting; PM2_Supporting；PP3
DUOX2	NM_014080.5	c.2290C>T	LP	PP3_Moderate; PM1; PM3

DUOX2	NM_014080.5	c.2654G>A	LP	PS3_Supporting; PP3_Supporting; PM2_Supporting; PM1; PP4
DUOX2	NM_014080.5	c.2654G>T	LP	PP3_Supporting; PM1; PM3, PP4
DUOX2	NM_014080.5	c.287C>T	LP	PS3_Supporting; PP3_Supporting; PM2_Supporting; PM3
DUOX2	NM_014080.5	c.2895-2898del	P	PM2_Supporting; PVS1
DUOX2	NM_014080.5	c.3175C>T	LP	PM2_Supporting; PM1; PM3, PP4
DUOX2	NM_014080.5	c.3232G>A	LP	PP3_Moderate; PS1； PP4
DUOX2	NM_014080.5	c.3251G>A	LP	PP3_Moderate; PM2_Supporting; PM1; PP4
DUOX2	NM_014080.5	c.3329G>A	LP	PS3_Supporting; PP3_Strong; PM1;
DUOX2	NM_014080.5	c.3391G>A	LP	PS3_Supporting; PP3_Moderate; PM1; PP4
DUOX2	NM_014080.5	c.3516-3531del	P	PM2_Supporting; PVS1
DUOX2	NM_014080.5	c.3540T>A	LP	PVS1_Unset; PP3_Strong; PM2_Supporting; PP4
DUOX2	NM_014080.5	c.3693+1G>T	P	PVS1_Unset; PM3
DUOX2	NM_014080.5	c.4027C>T	LP	PP3_Moderate; PS1； PP4
DUOX2	NM_014080.5	c.4408C>T	LP	PP3_Moderate; PM1; PM3
DUOX2	NM_014080.5	c.4537G>C	LP	PP3_Strong; PM2_Supporting; PM1;
DUOX2	NM_014080.5	c.602dupG	P	PVS1_Unset; PM2_Supporting; PM3
DUOX2	NM_014080.5	c.835G>A	LP	PP3_Moderate; PM2_Supporting; PM1; PP4
DUOXA2	NM_207581	c.148C>T	LP	PP3_Moderate; PM2_Supporting; PM1; PP4
DUOXA2	NM_207581	c.412-412delinsTA	P	PVS1_Unset; PM2_Supporting; PM3

Abbreviations: NGS, next generation sequencing; ACMG, American College of Medical Genetics; P, pathogenic; LP, likely pathogenic; VUS, variant of uncertain significance; B, benign; LB, likely benign

Table S5 The information of 119 patients with gene detection

Patients ID	Sex	Gene	Variations 1	Variations 2	TSH level at NBS, mIU/L
1	Male	TG	c.2581A>G(VUS)		205.41
1	Male	TSHR	c.463A>T		
2	Male	TG	c.4493C>T(VUS)		200.51
3	Female	TSHR	c.1580T>A	c.1580T>A	122.81
4	Female	TPO	c.425G>A	c.425G>A	140.81
5	Female	Negative			134.41
6	Female	TG	c.5386-5387delCAinsGCAATATCTT	c.5386-5387delCAinsGCAATATCTT	100
7	Male	DUOX2	c.3516-3531del	c.3516-3531del	95.1
8	Female	TPO	c.940C>T	c.940C>T	281
8	Female	NKX2-5	c.641_642insGCC		
8	Female	TG	c.7847A>T(VUS)		
8	Female	DUOX2	c.2182G>A		
9	Female	TG	c.4783delG	c.4783delG	100
10	Female	Negative			150
11	Male	TG	c.6517C>T	c.6517C>T	150
12	Male	TSHR	c.1291G>C	c.1291G>C	125
13	Female	TPO	c.2473T>C	c.2473T>C	144
14	Female	Negative			138
15	Female	TG	c.6707C>T	c.5921T>C(VUS)	200
16	Male	DUOX2	c.3516-3531del	c.3516-3531del	254
17	Female	TG	c.2359C>T	c.8119C>T	134
18	Female	TG	c.6707C>T	c.5386-5387delCAinsGCAATATCTT	111
19	Female	Negative			200
20	Female	TSHR	c.1736C>T	c.1736C>T	225
20	Female	DUOX2	c.1295G>A		
21	Female	TPO	c.1949G>A	c.1949G>A	77.4

22	Female	Negative			116
23	Male	TG	c.7239+4A>C(VUS)	c.4493C>T(VUS)	113
23	Male	NKX2-1	c.1054G>A		
24	Female	DUOX2	c.1300C>T	c.160+5G>A	100
25	Female	TPO	c.1246C>T	c.1246C>T	209
26	Female	PAX8	c.985T>C(VUS)		27
26	Female	SLC26A4	c.1790T>A		
26	Female	TSHR	c.154C>A		
27	Male	TSHR	c.394G>C		178
27	Male	GNAS	c.946G>C		
28	Female	Negative			230
29	Female	TPO	c.1949G>A	c.1949G>A	100
29	Female	DUOXA2	c.738C>T(VUS)		
30	Female	DUOX2	c.2102G>A(VUS)		262
31	Female	TPO	c.2677G>A		150
31	Female	TSHR	c.1349G>A		
32	Female	Negative			179
33	Male	TPO	c.872G>A	c.872G>A	280
34	Female	TPO	c.1229G>A	c.1229G>A	124
35	Male	TPO	c.1428_1448del	c.1428_1448del	325
36	Female	TG	c.1010G>A	c.1010G>A	147
36	Female	TG	c.4493C>T(VUS)	c.4493C>T(VUS)	
37	Male	Negative			30.2
38	Female	TG	c.7198A>G	c.7198A>G	74.3
38	Female	NKX2-1	c.1054G>A		
39	Female	TSHR	c.243-1G>C		92.31
39	Female	TG	c.4859C>T		
40	Male	DUOX2	c.3693+1G>T		100
41	Female	DUOX2	c.4027C>T	c.2048G>T	127.91
42	Female	Negative			189.51
43	Male	Negative			416.3

44	Male	DUOX2	c.2944C>G(VUS)		86.51
45	Female	SLC5A5	c.1572T>G	c.1572T>G	127.3
46	Male	DUOX2	c.3540T>A	c.3329G>A	81.41
47	Female	SLC26A4	c.*69C>A(VUS)	c.1708-18T>A(VUS)	42.01
47	Female	TG	c.4493C>T(VUS)	c.2963G>A	
48	Female	DUOX2	c.1588A>T	c.3329G>A	126.7
49	Female	DUOX2	c.2048G>T	c.2290C>T	99
50	Male	TPO	c.1781G>A	c.1905G>C	173.91
51	Female	DUOXA2	c.148C>T		110.01
51	Female	TG	c.3850C>T(VUS)		
52	Female	DUOX2	c.3175C>T		163.7
52	Female	TPO	c.256G>A		
53	Male	DUOX2	c.3329G>A	c.287C>T	79.4
54	Male	DUOX2	c.3329G>A	c.3329G>A	378.51
55	Female	DUOX2	c.1588A>T	c.3391G>A	100
56	Female	DUOX2	c.1588A>T	c.1588A>T	30.31
57	Male	DUOX2	c.3251G>A		100
58	Male	DUOX2	c.4537G>C		240.41
59	Female	Negative			258.7
60	Male	TSHR	c.1942A>T	c.350T>G	100
60	Male	DUOX2	c.835G>A		
61	Female	Negative			103.6
62	Female	GNAS	c.2514+31C>T(VUS)		204
63	Male	DUOX2	c.2654G>T		105.2
63	Male	DUOXA2	c.738C>T(VUS)		
63	Male	PAX8	c.1046C>A		
64	Male	TPO	c.612G>A	c.612G>A	172.1
64	Male	TPO	c.2578G>A	c.2578G>A	
65	Male	DUOX2	c.3693+1G>T	c.1268C>T	81.11
66	Male	DUOX2	c.1588A>T		192.05
66	Male	DUOXA2	c.412-412delinsTA		

67	Male	Negative			83.11
68	Female	DUOX2	c.3232G>A		311.5
69	Female	TPO	c.1428_1448del	c.1428_1448del	108.2
70	Male	TG	c.83C>T(VUS)		16.97
71	Female	TPO	c.551G>A		82.41
72	Male	DUOX2	c.1873C>T	c.1588A>T	100
73	Female	Negative			72.51
74	Male	DUOX2	c.4408C>T		40.41
75	Female	DUOX2	c.1588A>T	c.2654G>A	37.71
76	Male	TG	c.7847A>T(VUS)	c.7733G>A	63.21
76	Male	GNAS	c.334G>A(VUS)		
76	Male	TPO	c.2647C>T		
77	Female	Negative			150
78	Female	TG	c.2963G>A	c.2963G>A	99.41
78	Female	TG	c.5922G>A(VUS)	c.5922G>A(VUS)	
78	Female	GNAS	c.1729A>G(VUS)		
79	Female	TPO	c.1781G>A	c.1781G>A	221.61
79	Female	TPO	c.1905G>C	c.1905G>C	
79	Female	JAG1	c.574T>C		
79	Female	TSHR	c.154C>T		
80	Male	DUOX2	c.602dupG	c.2895-2898del	100
81	Female	DUOX2	c.2048G>T	c.4027C>T	55.41
82	Female	Negative			147.71
83	Female	TSHR	c.1195delA	c.1195delA	204.91
84	Male	Negative			153.01
85	Female	Negative			153.01
86	Male	POU1F1	c.*8C>T(VUS)		153.01
87	Male	DUOX2	c.2654G>A	c.3516-3531del	100
88	Male	DUOX2	c.3329G>A	c.1087A>G	63.92
89	Female	Negative			203.71
90	Female	Negative			203.71

91	Male	DUOX2	c.1873C>T(VUS)	c.2895-2898del	170.91
92	Male	DUOX2	c.1588A>T	c.3693+1G>T	100
93	Female	Negative			391.2
94	Female	Negative			138.71
95	Male	Negative			164.81
96	Male	Negative			100
97	Male	Negative			100
98	Female	Negative			426.21
99	Female	Negative			138.71
100	Female	TBL1X	c.482C>T(VUS)		115.71
101	Male	Negative			21.01
102	Male	Negative			154.81
103	Female	Negative			175.11
104	Male	TPO	c.1101_1110del	c.2387A>G	217.51
105	Female	Negative			27.71
106	Female	Negative			121.91
107	Female	DUOX2	c.1588A>T	c.1588A>T	100
108	Male	Negative			181.91
109	Female	Negative			208.31
110	Female	Negative			65.81
111	Female	Negative			65.81
112	Female	DUOX2	c.1588A>T	c.1310G>C	83.61
113	Male	TPO	c.71C>T(VUS)		291.31
114	Female	SLC26A4	c.1003T>C		424.91
114	Female	TPO	c.2306G>A		
115	Male	Negative			9.5
116	Female	DUOX2	c.3329G>A	c.602dupG	50.41
117	Female	TG	c.5928A>G		138.81
118	Female	Negative			26.21
119	Male	SLC5A5	c.206C>T	c.206C>T	122.41
